# Enhancing nitrogen use efficiency in agriculture by integrating agronomic practices and genetic advances

**DOI:** 10.3389/fpls.2025.1543714

**Published:** 2025-03-11

**Authors:** Aamir Ali, Nida Jabeen, Rasulov Farruhbek, Zaid Chachar, Azhar Ali Laghari, Sadaruddin Chachar, Nazir Ahmed, Shoaib Ahmed, Zhenping Yang

**Affiliations:** ^1^ College of Agriculture, Shanxi Agricultural University, Taigu, Jinzhong, China; ^2^ School of Communications and Information Engineering, Chongqing University of Posts and Telecommunication, Chongqing, China; ^3^ Andijan State Medical Institute, Department of Pharmaceutical Sciences, Andijan, Uzbekistan; ^4^ College of Agriculture and Biology, Zhongkai University of Agriculture and Engineering, Guangzhou, Guangdong, China; ^5^ College of Resources and Environment, Shanxi Agricultural University, Taigu, Shanxi, China; ^6^ College of Horticulture and Landscape Architecture, Zhongkai University of Agriculture and Engineering, Guangzhou, China; ^7^ Department of Agronomy, Sindh Agriculture University Campus, Umerkot, Pakistan; ^8^ Department of Agronomy, Faculty of Crop Production, Sindh Agriculture University, Tandojam, Pakistan

**Keywords:** nitrogen use efficiency, agronomic practices, genetic improvement, sustainable agriculture, soil health

## Abstract

Nitrogen is a critical nutrient for plant growth and productivity, but inefficiencies in its use in agriculture present both economic and environmental challenges. Enhancing nitrogen use efficiency (NUE) is essential for promoting sustainable crop production and mitigating the negative impacts of nitrogen loss, such as water pollution and greenhouse gas emissions. This review discusses various strategies aimed at improving NUE, with a focus on agronomic practices, genetic advancements, and integrated management approaches. Traditional agronomic methods, including split nitrogen application and the use of controlled-release fertilizers, are explored alongside precision agriculture techniques, which enable real-time adjustments to nitrogen application based on crop and soil conditions. Advances in genetics and biotechnology, such as conventional breeding, genetic modification, and genome editing, have contributed to the development of crop varieties with improved nitrogen uptake and assimilation. Additionally, the role of beneficial microbes, including nitrogen-fixing bacteria and mycorrhizal fungi, is highlighted as a natural means of enhancing nitrogen availability and reducing reliance on synthetic fertilizers. The review further emphasizes sustainable practices such as legume-based crop rotations, continuous cover cropping, and organic fertilization, which contribute to soil nitrogen enrichment and overall soil health. By combining these agronomic, genetic, and microbial strategies, a holistic nitrogen management approach can be achieved, maximizing crop yields while minimizing environmental impacts. This integrated strategy supports the development of resilient and sustainable agricultural systems, promoting long-term soil fertility and productivity.

## 1 Introduction

Nitrogen (N) is an essential nutrient that plays a vital role in plant growth and development. It is a fundamental component of amino acids, proteins, chlorophyll, and nucleic acids and is crucial for various physiological processes. In addition to photosynthesis, nitrogen is vital for protein synthesis, which supports enzyme function and structural integrity in plants (Wang et al., 2024). It also plays a key role in nitrogen assimilation, whereby plants convert inorganic nitrogen into organic forms, facilitating cell division and tissue development. Nitrogen is involved in energy metabolism through its role in the synthesis of ATP, aiding in nutrient transport and overall plant vigor (Wang et al., 2024). In agriculture, nitrogen is a key nutrient that significantly influences crop yield and quality. As plants cannot directly absorb atmospheric nitrogen, they depend on nitrogen available in the soil, mainly in the forms of nitrate (NO_3_⁻) and ammonium (NH_4_^+^), for their growth (Coskun et al., 2017). The impact of on agriculture is profound as it directly affects the productivity of staple crops such as rice, wheat, and maize. These crops require large amounts of nitrogen to achieve optimal growth and yield. However, the challenge lies in efficient management of nitrogen. While fertilizers have become a primary source of nitrogen for crops, the inefficiency in their use poses both economic and environmental issues (Yadav and Research, P. D, 2024). On average, crops absorb only approximately 50% of the nitrogen applied through fertilizers, with the reminder lost through processes such as volatilization, leaching, and denitrification​. These losses not only result in lower crop productivity but also contribute to environmental problems, including water pollution and greenhouse gas emissions (Anas et al., 2020). Therefore, enhancing NUE has become a critical objective in sustainable agriculture. By improving NUE, it is possible to maximize crop yields while minimizing the adverse effects of nitrogen loss in the environment (Hirel et al., 2011). This review explores the current practices, genetic approaches, and future prospects for optimizing nitrogen management in agriculture.

Despite its critical role in plant growth, the application of nitrogen in agriculture is fraught with inefficiency. On average, crops use only approximately 50% of the nitrogen applied through fertilizers, with the remainder lost to various processes such as volatilization, leaching, and denitrification​. This inefficiency not only limits crop productivity but also leads to significant economic losses for farmers (Fathi, 2022) Excessive nitrogen loss poses significant challenges, including environmental issues such as water pollution and greenhouse gas emissions. The environmental consequences of excessive nitrogen loss are alarming, highlighting the urgent need to enhance NUE in agriculture. When nitrogen is lost from agricultural fields, it often enters nearby water bodies as nitrate, causing eutrophication a process that leads to the overgrowth of algae and depletion of oxygen in water, severely affecting aquatic life. In addition, to aquatic ecosystems, nitrogen leaching into the soil can lead to soil acidification, disrupting nutrient availability and microbial activity, which ultimately degrades soil quality. Acidic soils can inhibit the growth of beneficial microorganisms and reduce nutrient cycling efficiency. Excessive nitrogen loss also negatively affects terrestrial ecosystems by altering plant community structure and reducing biodiversity. Certain nitrogen-loving plant species can dominate ecosystems, leading to a decline in species richness and changes in wildlife habitat quality. Additionally, nitrogen loss through denitrification results in the release of nitrous oxide (N_2_O), a potent greenhouse gas that contributes to climate change (Singh and Craswell, 2021). Agriculture is a major source of N_2_O emissions, accounting for a significant portion of global greenhouse gas emissions (Pan et al., 2022). These environmental impacts underscore the urgent need to improve NUE in agriculture.

An additional challenge in nitrogen management arises from the significant variability in soil properties, climatic conditions, crop species, and farming practices. These factors vary widely across regions and require tailored approaches rather than a uniform nitrogen management strategy. Furthermore, the excessive use of nitrogen fertilizers, often intended to boost yields, can result in soil degradation, nutrient imbalances, and diminished NUE over time. This over-reliance on fertilizers also contributes to long-term declines in soil health (Zhang et al., 2024). Therefore, achieving an optimal balance between high crop productivity and reduced environmental impact necessitates the adoption of integrated and site-specific nitrogen management strategies that consider local conditions.

This review aims to explore various strategies for enhancing NUE in agricultural systems to address both economic and environmental concerns. The focus is on integrating agronomic practices, genetic approaches, and advanced technologies to optimize nitrogen management. By reviewing current research, this paper highlights how conventional farming methods, precision agriculture, genetic modifications, and microbial interactions can be leveraged to improve (NUE) in crops. Furthermore, the review will examine future prospects for sustainable nitrogen management, including the potential shift toward legume-based crop rotations and the application of organic nitrogen sources (Sharma and Bali, 2018). The goal is to provide a comprehensive understanding of how these combined strategies can enhance crop productivity while reducing nitrogen losses, thereby promoting agricultural sustainability.

## 2 Nitrogen use efficiency

### 2.1 Definition and significance in agriculture

NUE is a measure of how effectively a plant utilizes available nitrogen to produce yield. It is often expressed as the ratio of output (crop yield or biomass) to input (amount of nitrogen applied). High NUE means that crops are efficiently converting the available nitrogen into growth, while low NUE indicates inefficiency, leading to nitrogen losses through leaching, volatilization, and denitrification (Chen et al., 2020) NUE is significant in agriculture for several reasons. First, it directly impacts crop productivity. Optimal nitrogen availability supports crucial plant processes like photosynthesis, protein synthesis, and overall growth, resulting in higher yields (Pan et al., 2022). Secondly, improving NUE can reduce the amount of nitrogen fertilizer required, leading to cost savings for farmers. Given that nitrogen fertilizers represent a major input cost in agriculture, efficient use is economically beneficial.

In addition, improving NUE is vital for environmental sustainability. Reducing nitrogen loss minimizes the environmental impact of agriculture, including water pollution from nitrate leaching, soil acidification, and greenhouse gas emissions. Therefore, enhancing NUE is a key strategy in sustainable farming practices, helping to balance the need for increased food production with the imperative to protect natural ecosystems.

However, several factors that influence NUE in crops, with soil properties, climate conditions, and fertilizer application methods play a critical role. Understanding these factors is key to developing strategies that optimize nitrogen use, thereby enhancing crop productivity and minimizing environmental impacts (Govindasamy et al., 2023) Soil properties are major determinants of NUE. Texture, organic matter content, pH, and microbial activity within the soil directly affect the storage, transformation, and availability of nitrogen to plants. Soils rich in organic matter serve as a reservoir of nitrogen and slowly release it in forms that are accessible to plants. However, sandy soils, owning to their large pore spaces, are more prone to nitrogen leaching, leading to a reduction in NUE. Soil pH also plays an essential role in acidic soils, microbial activity decreases, limiting the conversion of nitrogen into plant-usable forms. Furthermore, nitrogen may become fixed in insoluble compounds at low pH levels, reducing its availability to plants (Jaša et al., 2019). To address these issues, proper soil management practices, such as liming to adjust soil pH and adding organic matter to increase nutrient-holding capacity, can significantly enhance NUE.

Climate conditions: including temperature, rainfall, and humidity affect nitrogen dynamics in the soil and the ability of plants to absorb it. High rainfall can result in the leaching of nitrogen from the root zone, particularly in sandy soils, whereas drought conditions can limit nitrogen uptake due to reduced root activity and nutrient transport. Temperature fluctuations influence microbial processes like nitrification and denitrification, which affect the forms of nitrogen available for plant uptake. For instance, warmer temperatures can accelerate nitrogen mineralization, increasing the amount of plant-available nitrogen. In contrast, cooler temperatures may slow down microbial processes. Consequently, adopting climate-smart practices, such as adjusting nitrogen application timing and rates based on weather patterns, is crucial for optimizing NUE under varying climatic conditions (Novick et al., 2016) Fertilizer application methods, including source, rate, timing, and placement, are also vital in influencing NUE. The type of nitrogen fertilizer used (e.g., urea, ammonium nitrate, or slow-release fertilizers) affects the nitrogen availability and loss pathways. For example, slow-release fertilizers and nitrification inhibitors can enhance NUE by reducing nitrogen loss through leaching and volatilization. The rate of nitrogen application should match the crop’s specific growth requirements; over-application leads to nitrogen loss, whereas under-application limits plant growth. Soil testing and plant tissue analysis are useful tools for determining the optimal nitrogen application rate for specific crops and soil conditions. Timing the application of nitrogen to coincide with critical growth stages is equally important. Splitting nitrogen applications into multiple doses throughout the growing season can enhance plant uptake efficiency. Proper placement of fertilizers, such as banding or side-dressing, ensures that nitrogen remains in the root zone, thereby reducing losses (Maharjan et al., 2022). Additionally, employing precision agriculture tools, such as soil sensors and GPS-based application technologies, can further improve NUE by accurately applying nitrogen, both spatially and temporally, to meet plant needs.

Collectively, these factors highlight the complexity of managing nitrogen efficiently in agricultural systems (Miner et al., 2020). By optimizing soil properties, adjusting practices to suit climatic conditions, and employing advanced fertilizer application methods, farmers can significantly enhance NUE, resulting in higher crop productivity and reduced environmental impacts.

### 2.2 Strategies for enhancing NUE in rainfed and dryland systems

**Figure 1:**
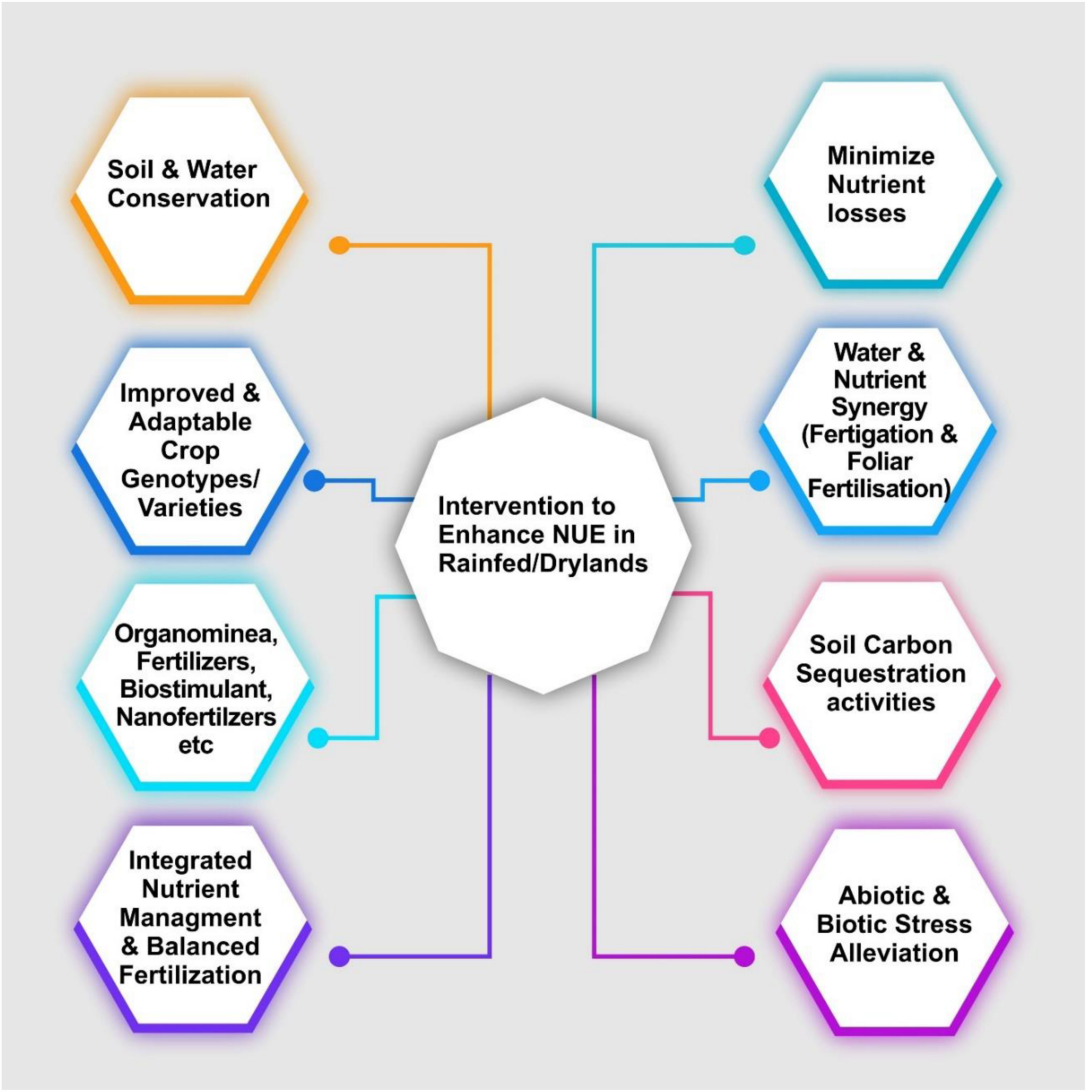
An overview of interventions to enhance Nutrient Use Efficiency (NUE) in rainfed and dryland systems. The strategies focus on soil and water conservation, adaptable crop varieties, balanced fertilization, and stress alleviation to support nutrient retention and utilization in water-limited environments.

The strategies depicted in **Figure 1** emphasize a comprehensive approach to improving NUE specifically tailored to the challenges of rainfed and dryland ecosystems. These interventions focus on optimizing nutrient utilization while mitigating environmental and physiological stress factors characteristic of water-limited agricultural systems. One primary strategy involves soil and water conservation measures, which are essential for preserving soil moisture and reducing nutrient leaching in dry environments. Techniques such as mulching, contour farming, and rainwater harvesting contribute to enhanced water retention, providing a stable foundation for nutrient availability in rainfed areas. Another critical intervention is the development of improved and adaptable crop genotypes and varieties. Selecting genotypes with higher resilience to nutrient-poor soils and drought conditions enhances plant performance under low input scenarios, allowing crops to utilize available nutrients more effectively. These varieties are also better suited to withstand the abiotic stressors inherent in dryland agriculture. The use of organ mineral fertilizers, bio stimulants, and nano fertilizers offers a diversified nutrient supply that supports plant growth under nutrient-deficient conditions. These inputs improve nutrient availability and soil health, with bio stimulants boosting root development and nano fertilizers providing controlled-release nutrient delivery. Integrated Nutrient Management (INM) and balanced fertilization further optimize nutrient application by tailoring it to crop requirements and growth stages, thereby maximizing nutrient uptake efficiency.

To prevent nutrient losses, strategies aimed at minimizing nutrient losses are incorporated, including precise nutrient application methods and minimizing runoff. The synergy between water and nutrient management through fertigation and foliar fertilization enhances nutrient assimilation by making nutrients available at critical developmental stages, reducing dependency on soil-bound nutrients. Soil carbon sequestration activities are also included in the framework, aiming to improve soil structure, increase organic matter, and enhance nutrient retention. Practices such as crop residue management and reduced tillage not only sequester carbon but also enrich soil health, providing long-term support for nutrient cycling in these systems. Finally, addressing abiotic and biotic stress alleviation is integral to improving NUE in drylands. Drought and pest resistance, achieved through targeted breeding or genetic modifications, reduce the likelihood of nutrient wastage due to stress-related plant responses, thus improving overall nutrient uptake and utilization efficiency.

These interventions represent a multidimensional strategy designed to enhance (NUE) in rainfed and dryland ecosystems. By integrating genetic, agronomic, and environmental approaches, this framework supports sustainable agriculture in resource-limited settings and aims to improve crop resilience, productivity, and environmental sustainability.

## 3 Agronomic practices for nitrogen management

### 3.1 Fertilizer Strategies

Optimizing (NUE) in agriculture depends heavily on effective fertilizer strategies. Traditional methods, such as split nitrogen application, combined with modern precision agriculture techniques, play a crucial role in improving nitrogen management. One of the most widely used traditional strategies for enhancing NUE is split application, where nitrogen fertilizer is applied in multiple doses throughout the growing season rather than in a single large application. This approach aligns nitrogen availability with the plant’s varying demand at different growth stages. By supplying nitrogen during critical phases such as early vegetative growth and flowering, split application helps reduce losses due to leaching, volatilization, and denitrification. For example, applying a portion of nitrogen at sowing and then supplementing during peak growth periods ensures that plants can access nitrogen when they need it most, thereby maximizing uptake and reducing environmental losses (Wang et al., 2018). In addition to split application, farmers often use controlled-release fertilizers that gradually release nitrogen into the soil. This slow-release mechanism matches the crop’s uptake pattern, enhances nitrogen availability over an extended period. Similarly, incorporating nitrification inhibitors into fertilizers can slow the conversion of ammonium to nitrate, minimizing leaching losses and improving NUE (Shanmugavel et al., 2023). These practices provide a straightforward means to enhance nitrogen efficiency in crop production when used judiciously.

Modern precision agriculture tools have further refined nitrogen management, allowing for site-specific and real-time adjustments to fertilizer application. Precision farming involves using technologies such as GPS-guided equipment, soil sensors, and remote sensing to assess soil nutrient levels, crop health, and environmental conditions (Argento et al., 2021). These technologies enable farmers to apply nitrogen more accurately, avoiding both under and over-application. For instance, variable-rate technology (VRT) allows farmers to adjust fertilizer application rates across a field based on soil nutrient variability. Areas with high soil nitrogen content receive less fertilizer, while areas with lower content receive more, optimizing overall nitrogen use.

However, despite the potential benefits, the adoption of precision tools is often limited by high costs, lack of technical expertise, and accessibility issues, particularly for small-scale farmers in developing regions. The upfront investment required for GPS-guided equipment and soil sensors can be prohibitive, and ongoing maintenance adds to the financial burden. Moreover, many farmers lack access to reliable internet connectivity, which is essential for the effective use of remote sensing and data-driven decision-making tools. Addressing these barriers requires developing low-cost precision technologies, government subsidies, and training programs to equip smallholder farmers with the skills needed to implement precision agriculture effectively (Argento et al., 2021). Collaborative efforts between research institutions, technology providers, and policymakers will be crucial in making precision agriculture more inclusive and accessible.

Another aspect of precision agriculture is the use of crop monitoring tools, such as drones and satellite imagery, to track plant growth and nitrogen status throughout the growing season. These tools provide valuable data on crop health and nutrient deficiencies, thereby enabling timely intervention. For example, by analyzing chlorophyll content in leaves, farmers can assess the nitrogen status of plants and make in-season adjustments to fertilizer application. This real-time management ensures that crops receive adequate nitrogen, maximizing yield while minimizing excess fertilizer use. Integrating traditional methods like split application with precision agriculture techniques can significantly enhance NUE (Mana et al., 2024). Such an integrated approach not only boosts crop productivity but also mitigates the environmental impact of nitrogen loss, promoting more sustainable agricultural practices.

### 3.2 Practical limitations of precision tools

While precision agriculture tools such as Variable Rate Technology (VRT), GPS-based applications, and soil sensors have shown significant potential in improving NUE, their practical adoption is often hindered by several limitations (Yadav et al., 2023). One primary challenge is the high cost associated with acquiring and maintaining precision equipment. Many small-scale farmers, particularly in developing countries, lack the financial resources to invest in advanced technologies, making it difficult for them to adopt these tools.

In addition to cost, the accessibility of precision tools is another barrier. Precision agriculture relies on digital infrastructure, including reliable internet connectivity and access to data analytics platforms, which may not be available in remote or underdeveloped regions (Goyal et al., 2023).Furthermore, the successful implementation of precision technologies requires technical expertise in data interpretation and equipment operation. Without proper training and support, farmers may struggle to utilize these tools effectively, limiting their potential benefits.

Another limitation is the scale of operation. Precision technologies are often more feasible for large-scale farms where the high initial investment can be justified by greater efficiency gains. In contrast, small-scale farmers may find it challenging to achieve similar returns on investment (Goyal et al., 2023). Addressing these limitations will require the development of cost-effective precision tools, government subsidies or incentives for technology adoption, and farmer training programs to build capacity and promote widespread use.

### 3.3 Alternative practices

Alternative practices in nitrogen management, such as no-till farming, cover cropping, and water management, provide sustainable ways to enhance NUE while reducing environmental impacts. No-till farming minimizes soil disturbance by avoiding traditional plowing, which helps maintain soil structure and organic matter content. This practice promotes the natural accumulation of organic nitrogen in the soil and reduces nitrogen losses through erosion and leaching. By preserving soil moisture and fostering beneficial microbial activity, no-till farming creates a conducive environment for steady nitrogen release and uptake by crops. Additionally, it can increase soil carbon content, enhancing the ability of the soil to retain nutrients, including nitrogen, thus improving NUE over time (Elrys et al., 2022). Cover crops, such as legumes, grasses, and clovers, are grown during off-season periods when the main crops are not actively cultivated. These crops act as natural nitrogen providers, particularly leguminous plants that can fix atmospheric nitrogen through symbiotic relationships with soil bacteria. Cover crops also help prevent soil erosion, reduce nutrient leaching, and increase organic matter content, all of which contribute to more efficient nitrogen cycling in the soil (Adetunji et al., 2020). When cover crops decompose, they release nitrogen in forms that are more accessible to subsequent crops, reducing the need for synthetic nitrogen fertilizers.

Water management is another critical factor in enhancing NUE. Efficient irrigation practices, such as drip irrigation or controlled irrigation schedules, ensure that water is applied directly to the root zone in the right amount, minimizing waterlogging and leaching of nitrogen. Over-irrigation often leads to the loss of nitrogen through runoff and leaching, particularly in sandy soils. By closely monitoring soil moisture levels and adjusting irrigation accordingly, farmers can create optimal conditions for nitrogen uptake by crops. Coupling water management with nitrogen application, known as fertigation, allows precise control over the timing and amount of nitrogen delivered, further enhancing NUE (Schaible and Aillery, 2013). Together, these alternative practices no-till farming, cover cropping, and water management complement traditional and precision agriculture strategies, creating an integrated approach to improving the NUE while promoting soil health and environmental sustainability.

## 4 Genetic approaches to enhance NUE

### 4.1 Breeding and biotechnology

Efforts in crop breeding and biotechnology have made significant contributions to enhancing NUE by improving nitrogen uptake, assimilation, and utilization in plants. These genetic approaches aim to develop crop varieties that can thrive with lower nitrogen inputs while maintaining high yields, thereby promoting both economic and environmental sustainability in agriculture.

#### 4.1.1 Traditional breeding

Conventional breeding programs have focused on selecting crop varieties with higher NUE. These programs utilize the natural genetic diversity within crops to identify traits linked to efficient nitrogen uptake and utilization. By selecting and cross-breeding high-performing lines, researchers have been able to develop varieties that exhibit traits such as enhanced root architecture, increased nitrogen assimilation, and optimized nitrogen partitioning within the plant (Lamichhane and Thapa, 2022). For example, breeding efforts in cereal crops like rice, wheat, and maize have resulted in varieties that are better adapted to low-nitrogen conditions, allowing for reduced fertilizer application without compromising yield.

In addition to conventional breeding, genetic modification, and genome editing, synthetic biology has emerged as a promising technology for enhancing NUE in crops. Synthetic biology involves designing and constructing novel genetic circuits and metabolic pathways to optimize nitrogen uptake, assimilation, and utilization in plants (Liu et al., 2023). Unlike traditional genetic approaches, which rely on modifying existing genes, synthetic biology allows for the creation of entirely new biological functions.

One example of synthetic biology in nitrogen optimization is the engineering of nitrogen-fixing capabilities in non-leguminous crops. While legumes naturally form symbiotic relationships with nitrogen-fixing bacteria, most staple crops, such as rice, wheat, and maize, do not possess this ability. Researchers are exploring synthetic approaches to transfer nitrogen-fixation genes from bacteria into these crops, potentially reducing the need for synthetic nitrogen fertilizers (Bennett et al., 2023). Early experimental work has demonstrated the feasibility of expressing bacterial nitrogenase enzymes in plant chloroplasts, paving the way for further advancements.

Another promising application of synthetic biology is the development of crops with enhanced nitrogen sensing and signaling pathways. By constructing synthetic regulatory networks, researchers can create plants that dynamically adjust their nitrogen uptake and metabolism in response to changing environmental conditions (Choi et al., 2023). This fine-tuned nitrogen management reduces waste and improves overall NUE.

Despite its potential, the application of synthetic biology in agriculture is still in its early stages and faces several challenges, including regulatory hurdles, public acceptance, and the technical complexity of designing robust and stable genetic circuits. Continued research and collaboration between synthetic biologists, agronomists, and policymakers will be critical to overcoming these barriers and realizing the full potential of synthetic biology in sustainable nitrogen management.

#### 4.1.2 Genetic modification and biotechnology

Advances in molecular genetics and biotechnology have enabled more targeted modifications to enhance NUE. One approach involves the overexpression of genes involved in nitrogen transport and assimilation. For instance, researchers have modified plants to overexpress nitrate transporter genes, leading to improved nitrogen uptake and assimilation. In rice, overexpression of the nitrate transporter gene OsNRT1.1B has been shown to increase nitrogen uptake, resulting in enhanced growth and grain yield. Similarly, manipulating the expression of genes encoding key enzymes in the nitrogen assimilation pathway, such as glutamine synthetase and nitrate reductase, has demonstrated potential in improving the efficiency of nitrogen conversion within plants (Abdul Aziz et al., 2022). In addition to nitrogen transporters and assimilation enzymes, genetic engineering has targeted the regulation of nitrogen-responsive pathways. By modifying signaling pathways that regulate nitrogen metabolism, plants can be engineered to optimize nitrogen uptake and utilization based on environmental conditions (Gaudinier et al., 2018). For example, introducing or modifying regulatory genes that control root development can lead to an increased root surface area, allowing for more effective nitrogen absorption from the soil.

#### 4.1.3 Genome editing technologies

Recent advancements in genome editing technologies, such as CRISPR/Cas9, have further accelerated efforts to enhance NUE. These tools allow for precise modifications of genes related to nitrogen metabolism, transport, and signaling, facilitating the development of crops with improved nitrogen uptake capabilities (Pacesa et al., 2024). For instance, genome editing can be used to knock out genes that negatively regulate nitrogen uptake or to introduce beneficial mutations that enhance the plant’s ability to absorb and utilize nitrogen efficiently.

#### 4.1.4 Marker-assisted and genomic selection

Marker-assisted selection (MAS) and genomic selection (GS) are modern breeding techniques that leverage genetic markers associated with high NUE traits. MAS involves selecting plants with desirable genetic markers linked to efficient nitrogen use, while GS utilizes genome-wide marker data to predict and select plants with the highest potential for NUE improvement (Degen and Müller, 2023). These techniques accelerate the breeding process by enabling the selection of plants with optimal nitrogen uptake and utilization characteristics without the need for extensive field testing.

The integration of traditional breeding, genetic modification, and modern biotechnological approaches have significantly contributed to the development of crop varieties with enhanced NUE (Abdul Aziz and Masmoudi, 2024). These advancements not only enable higher crop productivity with reduced nitrogen inputs but also mitigate the environmental impacts associated with excessive nitrogen fertilization.

### 4.2 Recent advances

Recent advances in genomics have revolutionized the identification of genes associated with NUE, providing new insights into the molecular mechanisms underlying nitrogen uptake, transport, assimilation, and signaling in crops. High-throughput sequencing technologies, functional genomics, and systems biology approaches have enabled researchers to identify key genes and regulatory networks involved in nitrogen metabolism, facilitating the development of crops with enhanced NUE (Adeoluwa et al., 2022) One significant advancement is the use of genome-wide association studies (GWAS) and quantitative trait loci (QTL) mapping to uncover genes and genetic markers linked to NUE. These studies examine the genetic diversity present in crop populations, identifying loci associated with traits such as nitrogen uptake efficiency, assimilation, and nitrogen partitioning within the plant. For example, in rice, several QTLs related to NUE have been identified, revealing genes that play crucial roles in nitrate uptake, transport, and assimilation (He and Gai, 2023). Breeding programs can use these genetic markers to select for high-NUE varieties through marker-assisted selection (MAS), speeding up the development of crops that require less nitrogen input while maintaining high yields.

Transcriptome analysis has also contributed to the identification of NUE-related genes. By analyzing the expression patterns of genes in response to different nitrogen levels, researchers have identified key nitrogen-responsive genes that regulate various aspects of nitrogen metabolism. For instance, transcriptomic studies in crops like wheat and maize have revealed the upregulation of nitrate transporters and nitrogen assimilation enzymes under low-nitrogen conditions, providing targets for genetic modification to enhance NUE (Wang et al., 2024) CRISPR/Cas9 genome editing technology has further facilitated the functional analysis of NUE-associated genes. Using this precise gene-editing tool, researchers can knock out or modify specific genes to assess their impact on nitrogen uptake and assimilation (Aljabali et al., 2024). This approach has been used to manipulate genes encoding nitrate transporters, such as NRT1.1, in crops like rice, resulting in improved nitrogen uptake and growth performance. Moreover, the targeted modification of regulatory genes involved in nitrogen signaling pathways has enabled the fine-tuning of nitrogen metabolism, enhancing overall NUE.

Additionally, the use of genomic selection (GS) in breeding programs allows for the prediction of NUE traits based on genome-wide marker data. GS uses high-density genetic markers to predict the genetic potential of plants, enabling breeders to select for high-NUE individuals more efficiently. This approach accelerates the development of NUE-improved crop varieties by reducing the time and resources required for traditional breeding methods (Alemu et al., 2024) Overall, the integration of genomics into nitrogen management research has provided a wealth of information on the genetic basis of NUE. These findings offer valuable targets for genetic modification, breeding, and molecular marker development, paving the way for the creation of crop varieties that can thrive in low-nitrogen environments, thus contributing to more sustainable agricultural practices.

### 4.3 CRISPR/Cas-mediated gene editing for improved (NUE) in Crops

**Figure 2:**
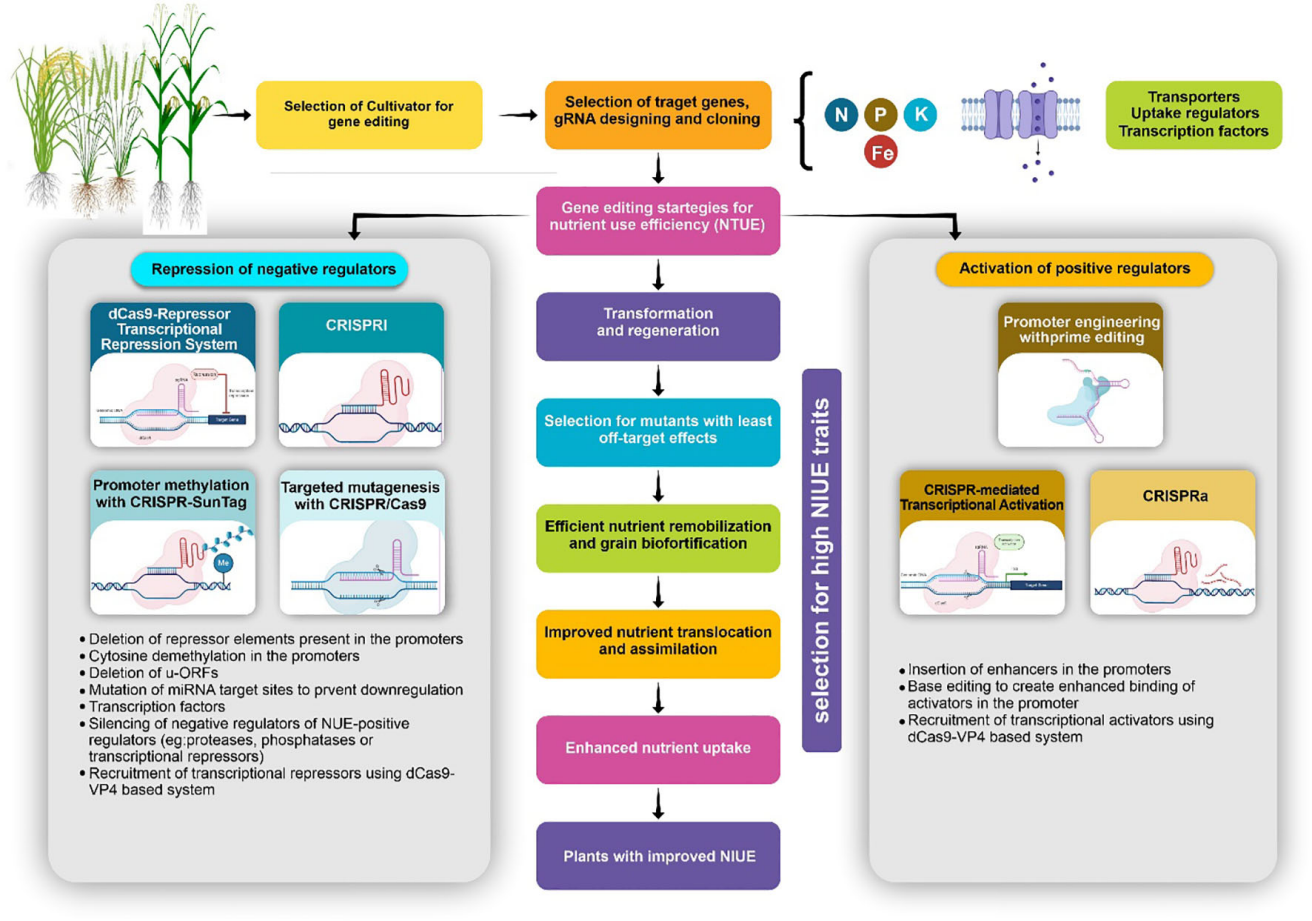
Demonstration of a gene editing approach targeting negative and positive regulators of NUE in crops. Negative regulators are suppressed using tools like CRISPRi and promoter methylation, while positive regulators are activated through CRISPRa and promoter engineering. The process aims to develop plants with optimized nutrient uptake, translocation, and assimilation for improved NUE traits.

The **Figure 2** illustrates a comprehensive gene editing strategy aimed at enhancing NUE in crops through targeted activation and repression of regulatory genes. This approach begins with the selection of cultivars and the design of guide RNAs (gRNAs) specific to genes involved in nutrient uptake and transport, focusing on essential elements such as nitrogen (N), phosphorus (P), potassium (K), and iron (Fe). The gene editing framework is divided into two core pathways: repression of negative regulators and activation of positive regulators. For repression, tools like the dCas9-repressor system, CRISPR interference (CRISPRi), promoter methylation (CRISPR-Sun Tag), and targeted mutagenesis via CRISPR/Cas9 are employed. These methods aim to silence genes that inhibit NUE by mechanisms including deletion of repressor elements, cytosine demethylation, and repression of transcriptional repressors. This suppression of negative regulators supports higher nutrient assimilation.

On the activation side, the strategies focus on upregulating beneficial genes. Techniques such as promoter engineering with prime editing, CRISPR-mediated transcriptional activation, and CRISPRa systems enhance the expression of genes involved in nutrient uptake and assimilation by inserting enhancers and creating promoter-specific modifications. This activation is directed towards genes that facilitate efficient nutrient transport and utilization in plants. The process involves multiple stages: transformation and regeneration, selection for minimal off-target mutations, improved nutrient remobilization, and optimization of nutrient translocation. These steps collectively aim to foster plants with enhanced nutrient uptake capabilities, improved grain biofortification, and ultimately, superior NUE traits. This multi-faceted gene editing approach shows potential for developing crops with robust nutrient efficiency, addressing agricultural demands for sustainable crop production.

## 5 Environmental impacts

### 5.1 Reducing losses

Improved nitrogen management practices play a critical role in minimizing nitrogen losses and mitigating their environmental impact. Nitrogen loss from agricultural fields primarily occurs through volatilization, leaching, surface runoff, and denitrification. These processes lead to water pollution, soil degradation, and the release of greenhouse gases, including (N_2_O), a potent contributor to climate change. Strategies such as split fertilizer application, controlled-release fertilizers, and precision agriculture can significantly reduce these losses. By optimizing NUE through agronomic, genetic, and precision agriculture practices, these losses can be significantly reduced, promoting environmental sustainability (Elrys et al., 2023). One of the most effective strategies for reducing nitrogen loss is split fertilizer application. By applying nitrogen in smaller, timed doses throughout the growing season rather than a single large application, plants can absorb the nutrient more efficiently (Aroonsrimorakot and Laiphrakpam, 2023). This approach reduces the risk of leaching, especially during periods of heavy rainfall, and minimizes nitrogen volatilization. For example, the timely application of nitrogen during critical growth stages ensures that it is available to the crop when demand is highest, decreasing the likelihood of nitrogen accumulating in the soil and subsequently being lost to the environment (Ma et al., 2023). The use of controlled-release fertilizers and nitrification inhibitors also plays a crucial role in reducing nitrogen losses. Controlled-release fertilizers gradually release nitrogen into the soil, aligning with the plant’s nutrient uptake pattern. This slow-release mechanism helps prevent nitrate leaching into groundwater and (N_2_O) emissions, a potent greenhouse gas. Nitrification inhibitors work by slowing down the conversion of ammonium to nitrate, reducing the risk of leaching and denitrification, which contributes to N_2_O emissions (Vejan et al., 2021). These advanced fertilizer products enhance NUE by ensuring nitrogen remains in the root zone for a longer period, allowing for more effective plant absorption.

Precision agriculture techniques, such as soil nutrient mapping, GPS-guided fertilizer application, and real-time crop monitoring, further contribute to reducing nitrogen losses. By using soil sensors and remote sensing technologies, farmers can tailor nitrogen applications to the specific needs of different field zones. This targeted application prevents over-fertilization, reduces surface runoff, and minimizes the risk of nitrogen entering nearby water bodies, thus reducing eutrophication. Additionally, real-time monitoring of crop nitrogen status allows for timely adjustments in fertilizer application, ensuring that crops receive adequate nutrition without excess nitrogen remaining in the soil (Raza et al., 2023). Cover cropping and crop rotation, particularly with legumes, are other effective practices for reducing nitrogen losses. Leguminous crops naturally fix atmospheric nitrogen, enriching the soil with organic nitrogen and reducing the need for synthetic fertilizers. Planting cover crops during off-seasons helps to retain soil nitrogen, prevent erosion, and reduce surface runoff, thus minimizing nitrogen loss to the environment. When these cover crops decompose, they release organic nitrogen slowly, providing a steady nutrient source for subsequent crops and reducing the reliance on chemical fertilizers (Scavo et al., 2022). Genetic approaches to improving NUE in crops also indirectly contribute to reducing environmental nitrogen loss. By developing crop varieties that can thrive with lower nitrogen inputs, the total amount of nitrogen fertilizer applied can be reduced, consequently decreasing the potential for nitrogen runoff, leaching, and volatilization (Hu et al., 2023). These high-NUE crops optimize nitrogen uptake, assimilation, and partitioning, ensuring that more of the applied nitrogen is utilized for growth and yield.

Integrated nitrogen management strategies including split fertilizer application, controlled-release products, precision agriculture, and genetic improvements can significantly reduce nitrogen losses from agricultural systems. These practices not only enhance crop productivity but also protect soil and water resources, reduce greenhouse gas emissions, and promote long-term environmental sustainability.

### 5.2 Soil health

Improved nitrogen management practices not only enhance NUE but also have a positive impact on soil health and fertility. When nitrogen is applied effectively, it contributes to building and maintaining soil organic matter, improving soil structure, and promoting beneficial microbial activity, all of which are essential for long-term soil fertility (Hu et al., 2023). One key benefit is the increase in soil organic matter. Proper nitrogen application promotes vigorous plant growth, resulting in greater root biomass and crop residues that return organic matter to the soil. This organic matter, when decomposed by soil microorganisms, forms humus, a stable form of organic carbon that enhances soil structure. Improved soil structure increases water retention, aeration, and nutrient availability, creating a more favorable environment for plant roots and soil biota (Dynamics of soil organic matter in tropical ecosystems, 1990). This increase in soil organic content also serves as a slow-release reservoir of nutrients, including nitrogen, which further supports plant growth over time.

Enhanced nitrogen management can also lead to a diverse and active soil microbial community. When nitrogen is applied efficiently, it minimizes the risk of nitrogen loss through leaching and volatilization, reducing the likelihood of soil acidification and nutrient imbalances. A balanced soil environment supports a rich microbial community, including nitrogen-fixing bacteria, which convert atmospheric nitrogen into forms that plants can use. These beneficial microbes play a critical role in nutrient cycling, decomposition of organic matter, and suppression of soil-borne diseases. By maintaining an optimal balance of soil nutrients through responsible nitrogen management, soil health and fertility are preserved, ultimately supporting sustainable crop production (Hayashi, 2022). Additionally, practices such as cover cropping, and the use of organic fertilizers improve soil fertility by enriching the soil with organic nitrogen and promoting nutrient cycling. Leguminous cover crops, for example, fix atmospheric nitrogen, adding organic nitrogen to the soil when they decompose. This process not only boosts soil fertility but also reduces the need for synthetic nitrogen fertilizers, lowering the risk of soil degradation associated with over-fertilization (Liu et al., 2023). The addition of organic matter through cover crops also improves soil texture, aeration, and water-holding capacity, contributing to a healthier and more resilient soil system.

Incorporating these soil-enhancing practices into nitrogen management strategies not only optimizes crop productivity but also ensures the long-term health and fertility of agricultural soils (Javed et al., 2022). This holistic approach to nitrogen management helps sustain soil ecosystems, promotes biodiversity, and supports the continued availability of nutrients, leading to more sustainable agricultural production.

## 6 Integrated management and future prospects

### 6.1 Combining agronomy and genetics

Improving NUE in agriculture requires an integrated approach that combines agronomic practices with genetic advancements. This synergy enables the development of crop production systems that optimize nitrogen uptake, reduce environmental impacts, and enhance soil health (Doyeni et al., 2023). Agronomic practices like precision agriculture, split fertilizer application, controlled-release fertilizers, and soil health management are foundational for improving NUE (Mohamed Selim, 2021). These strategies ensure that nitrogen is applied in the right amount, at the right time, and in the right place to match crop requirements. However, relying solely on these practices has limitations, as environmental factors such as soil type, climate, and weather patterns can still result in nitrogen losses.

On the other hand, genetic approaches involve breeding crop varieties that can efficiently utilize available nitrogen. These include selection for traits such as improved root architecture, enhanced nitrogen uptake and assimilation, and stress tolerance. Advances in genomics and molecular biology have facilitated the identification of genes and regulatory pathways involved in nitrogen metabolism, enabling the development of high-NUE crop varieties through techniques like marker-assisted selection and genome editing (Richard et al., 2022). By integrating agronomic practices with genetic approaches, it is possible to maximize the benefits of both strategies. For example, precision agriculture techniques can be used to manage nitrogen application in real-time, while genetically enhanced crops can make the most efficient use of the applied nitrogen. Crops bred for superior root systems can access nitrogen from deeper soil layers, reducing the need for frequent fertilizer applications (Bailey-Serres et al., 2019). Additionally, by selection traits that enhance nitrogen assimilation, crops can convert more absorbed nitrogen into biomass, resulting in higher yields with lower nitrogen inputs.

The integration of these strategies allows for more adaptive and resilient cropping systems. For instance, crops with improved genetic traits for nitrogen uptake can better cope with environmental stresses such as drought, which can limit the effectiveness of nitrogen fertilizers. In turn, agronomic practices such as optimizing irrigation and soil health management support the performance of these genetically enhanced crops, creating a feedback loop that promotes sustainable agricultural productivity (Acevedo et al., 2020). In the future, developing decision-support tools that incorporate both agronomic and genetic data will be crucial (Zhai et al., 2020). These tools can provide farmers with tailored recommendations on nitrogen management based on real-time field conditions and the specific genetic characteristics of their crops. By leveraging both agronomic practices and genetics, farmers can achieve higher NUE, reduce costs, and minimize the environmental impact of agricultural production, paving the way for a more sustainable future.

### 6.2 Microbial interactions

Beneficial soil microbes, including nitrogen-fixing bacteria and mycorrhizal fungi, play a crucial role in enhancing nitrogen uptake and improving NUE in plants (Dong et al., 2021). For instance, field trials using Rhizobium inoculants in legumes, such as soybeans and chickpeas, have demonstrated significant yield improvements of up to 25% compared to non-inoculated controls. Similarly, studies in India and Brazil have shown that co-inoculation with Azospirillum and phosphate-solubilizing bacteria enhances nitrogen and phosphorus uptake, resulting in increased maize yields under low-input farming systems.

However, the nitrogen fixing bacteria one of the most well-known groups of beneficial microbes is the rhizobia bacteria, which form symbiotic associations with leguminous plants. Rhizobia colonize plant roots, forming structures called nodules, where they convert atmospheric nitrogen (N_2_) into ammonium (NH_4_^+^) through a process known as biological nitrogen fixation. The ammonium is then available for plant uptake, providing a natural and sustainable source of nitrogen (Mahmud et al., 2020). This symbiosis significantly reduces the reliance on synthetic nitrogen fertilizers in legume-based cropping systems and improves soil fertility through the addition of organic nitrogen. In non-leguminous crops, free-living nitrogen-fixing bacteria, such as *Azotobacter* and *Azospirillum*, can also enhance nitrogen availability in the rhizosphere, further contributing to plant growth.

Microbial inoculants, such as biofertilizers containing nitrogen-fixing bacteria (e.g., Rhizobium, Azospirillum) and mycorrhizal fungi, are increasingly being used to enhance nitrogen availability and uptake. One notable example is the use of mycorrhizal biofertilizers in wheat fields in semi-arid regions, where mycorrhizal inoculation led to a 15% reduction in nitrogen fertilizer use without compromising yield. Additionally, biofertilizers based on Azotobacter have been shown to improve nitrogen fixation in non-leguminous crops, leading to higher nitrogen content in soils and better overall crop performance (Vandana et al., 2017). These biofertilizers can be applied to seeds, soil, or plant roots, boosting the population of beneficial microbes in the rhizosphere. They not only enhance NUE but also contribute to soil health by promoting microbial diversity. The inoculation of crops with these microbes has shown promising results in improving NUE, especially in low-nitrogen environments.

The integration of beneficial microbes into nitrogen management strategies offers a sustainable approach to enhancing NUE. By promoting symbiotic nitrogen fixation and improving nutrient absorption (Sahu et al., 2018), these microbes reduce the dependency on chemical fertilizers, mitigate nitrogen losses, and support soil health, thereby contributing to more resilient and sustainable agricultural systems.

Moreover, Arbuscular mycorrhizal (AM) fungi are another group of beneficial microbes that enhance nitrogen uptake. These fungi form symbiotic relationships with plant roots, extending their hyphal networks into the soil. This extensive network increases the root surface area, improving the plant’s access to soil nutrients, including nitrogen. Mycorrhizal fungi are particularly effective in absorbing immobile nutrients like ammonium and organic nitrogen compounds, facilitating their transfer to the host plant. In exchange, the plant provides the fungi with carbohydrates (Parihar et al., 2020). This symbiotic interaction not only enhances nitrogen uptake but also improves the overall nutrient status and health of the plant, promoting better growth and yield.

### 6.3 Sustainable practices

Promoting long-term sustainability in agriculture requires integrating practices that enhance NUE while preserving soil health and minimizing environmental impacts. One of the key strategies for future sustainable nitrogen management is legume-based crop rotations. Leguminous plants, such as soybeans, clover, and alfalfa, form symbiotic relationships with nitrogen-fixing rhizobia bacteria in their root nodules. This biological nitrogen fixation not only provides a direct nitrogen source for the legumes but also enriches the soil with organic nitrogen. When leguminous crops are rotated with non-leguminous crops, such as cereals, the following crops benefit from the residual nitrogen left in the soil. This practice reduces the need for synthetic nitrogen fertilizers, enhancing overall NUE and reducing the risk of nitrogen loss to the environment (Trang et al., 2019). Additionally, legume rotations contribute to improved soil structure, increased organic matter, and a more diverse soil microbial community, all of which are essential for sustainable agriculture.

Continuous cover cropping is another sustainable practice that complements legume rotations. Cover crops, grown during the off-season, help retain soil nitrogen and prevent leaching. Sustainable practices, such as legume-based crop rotations, continuous cover cropping, and organic fertilization, contribute to nitrogen cycling and improve soil fertility, reducing the need for synthetic nitrogen fertilizers (Garland et al., 2021). When these cover crops are incorporated into the soil as green manure, they provide a slow-release source of nitrogen for the subsequent crop, supporting more efficient nutrient cycling.

In addition to crop rotations and cover cropping, the use of organic fertilizers like compost, manure, and green manure offers a sustainable alternative to synthetic nitrogen fertilizers. Organic fertilizers release nitrogen gradually as they decompose, aligning more closely with the nutrient uptake patterns of crops (Boschiero et al., 2023; Gamage et al., 2023). This slow-release mechanism reduces the risk of nitrogen loss through volatilization and leaching, resulting in improved NUE. Moreover, organic fertilization contributes to enhancing soil health by increasing microbial diversity, improving soil structure, and enriching soil organic matter.

By integrating these sustainable practices, such as legume-based rotations, continuous cover cropping, and organic fertilization, with advanced agronomic techniques like precision agriculture and the use of genetically improved crop varieties, a holistic nitrogen management strategy can be developed (Shah et al., 2021). This integrated approach ensures a more balanced nitrogen cycle, supports high crop productivity, and minimizes environmental impacts, thereby paving the way for more resilient and sustainable agricultural systems.

## 7 Research gaps and future directions

While significant progress has been made in enhancing NUE through agronomic practices, genetic advancements, and precision agriculture, several research gaps remain. One critical area is the long-term impact of integrated nitrogen management strategies on soil health and biodiversity, particularly under varying climatic conditions. More research is needed to understand the synergies between genetic improvements and microbial interactions in improving NUE across diverse cropping systems.

Furthermore, while precision agriculture technologies have shown promise, their adoption is limited by cost and accessibility, especially in low-income regions. Future studies should focus on developing cost-effective precision tools and decision support systems that can be widely implemented by smallholder farmers. Another gap lies in the comprehensive assessment of the environmental trade-offs associated with various nitrogen management strategies. This includes quantifying the life-cycle impacts of advanced fertilizers and biofertilizers on greenhouse gas emissions and water quality. Finally, breeding programs for high-NUE crops need to incorporate advanced genomic tools such as CRISPR/Cas genome editing, combined with field-level evaluations to ensure that these genetically improved crops perform well under real-world conditions. Addressing these research gaps will be essential for promoting sustainable nitrogen management and enhancing global food security.

## 8 Conclusion

In conclusion, improving NUE is crucial for addressing both the economic and environmental challenges associated with nitrogen application in agriculture. This review has explored a range of strategies to enhance NUE, including traditional agronomic practices such as split nitrogen application and controlled-release fertilizers, as well as precision agriculture techniques that optimize fertilizer use based on real-time soil and crop conditions. Advances in genetics and biotechnology, including conventional breeding, genetic modification, and genome editing, have significantly contributed to the development of crop varieties with enhanced nitrogen uptake and assimilation. The role of beneficial microbes, particularly nitrogen-fixing bacteria and mycorrhizal fungi, has been highlighted as a natural and sustainable method to improve nitrogen availability in soils, reducing the reliance on synthetic fertilizers. Sustainable agricultural practices, such as legume-based crop rotations, continuous cover cropping, and organic fertilization, are essential for enriching soil nitrogen and maintaining soil health. By integrating these agronomic, genetic, and microbial strategies, a holistic nitrogen management approach can be achieved. This integrated strategy not only maximizes crop yields but also minimizes environmental impacts, fostering the development of more resilient and sustainable agricultural systems. Ultimately, this approach supports long-term soil fertility and productivity, contributing to global food security while safeguarding environmental health.
